# Polymeric nanocarrier-based adjuvants to enhance a locally produced mucosal coryza vaccine in chicken

**DOI:** 10.1038/s41598-024-65267-y

**Published:** 2024-07-03

**Authors:** Hazem M. Ibrahim, Gina M. Mohammed, Rafik Hamed Sayed, Hisham A. Elshoky, Marwa M. Ahmed, Marwa Fathy El Sayed, Shaimaa Abdelall Elsaady

**Affiliations:** 1 Veterinary Serum and Vaccine Research Institute (VSVRI), Agricultural Research Center (ARC), Cairo, Egypt; 2 Central Laboratory for Evaluation of Veterinary Biologics (CLEVB), Agricultural Research Center (ARC), Cairo, Egypt; 3 Nanotechnology and Advanced Materials Central Lab, Agricultural Research Center, Giza, Egypt; 4 Regional Center for Food and Feed, Agricultural Research Center, Giza, Egypt

**Keywords:** Iron oxide, Oleyl chitosan, Silicon dioxide, Infectious coryza, Nanomaterials, *A. Paragallinarum*, Nanovaccine, Immunology, Microbiology, Diseases, Materials science, Nanoscience and technology

## Abstract

Infectious coryza (IC) is an acute upper respiratory disease of chicken caused by *Avibacterium* (A.) *paragallinarum*. This disease results in an increased culling rate in meat chicken and a marked decrease in egg production (10% to more than 40%) in laying and breeding hens. Vaccines were first used against IC and effectively controlled the disease. Nanotechnology provides an excellent way to develop a new generation of vaccines. NPs have been widely used in vaccine design as adjuvants and antigen delivery vehicles and as antibacterial agents; thus, they can be used as inactivators for bacterial culture. In this research, the antibacterial effects of several nanoparticles (NPs), such as silicon dioxide with chitosan (SiO_2_-CS), oleoyl-chitosan (O.CS), silicon dioxide (SiO_2_), and iron oxide (Fe_3_O_4_), on *A. paragallinarum* were studied. Additionally, different *A. paragallinarum* vaccines were made using the same nanomaterials at a concentration of 400 µg/ml to help control infectious coryza disease in chicken. A concentration of 400 µg/ml of all the NPs tested was the best concentration for the inactivation of *A. paragallinarum*. Additionally, this study showed that the infectious coryza vaccine adjuvanted with SiO_2_ NPs had the highest immune response, followed by the infectious coryza vaccine adjuvanted with Fe_3_O_4_ NPs, the infectious coryza vaccine adjuvanted with SiO_2_-CS NPs, and the infectious coryza vaccine adjuvanted with O.CS NPs in comparison with the infectious coryza vaccine adjuvanted with liquid paraffin (a commercial vaccine).

## Introduction

Infectious coryza (IC) is a highly contagious bacterial disease caused by *Avibacterium paragallinarum* (*A. paragallinarum*) that primarily affects the upper respiratory tract of chicken. It is prevalent in chicken of all ages and is particularly common in intensive chicken farms, including large-scale egg production complexes and breeding farms, leading to decreased egg production^1,2^. The disease does not confer solid immunity, and adult chicken infected with IC do not pass immunity to their offspring through the egg. Vaccination is crucial for preventing and controlling infectious coryza infection, and the use of killed *A. paragallinarum* bacterin alone or in combination with bacterin containing other organisms is widely used for prevention^3^. Nevertheless, there is a need for enhancements to develop a more efficacious vaccine, necessitating the exploration of new and superior adjuvants^4^. Furthermore, advancements in vaccination involve the investigation of alternative methods, including painless and efficient needle-free approaches such as intranasal or oral administration. Administering vaccinations directly at the site of infection is known to boost mucosal immunity significantly. This approach proves especially beneficial for respiratory conditions like infectious coryza^5^. In recent years, nanomaterials have gained attention as potential adjuvants and inactivators in vaccine formulations. Among these nanomaterials, oleyl chitosan (O.CS), silicon dioxide (SiO_2_), and iron oxide (Fe_3_O_4_) have demonstrated promising properties for enhancing the immune response and improving vaccine efficacy against *A. paragallinarum*. Oleyl chitosan is a derivative of chitosan and is a biocompatible and biodegradable polysaccharide derived from chitin. O.CS possesses amphiphilic properties by combining the hydrophilicity of chitosan with the hydrophobicity of oleyl groups. The amphiphilic nature of O.CS facilitates efficient encapsulation and diverse antigen delivery, stimulating immunomodulatory effects and promoting robust immune responses. Its adaptability for various vaccine formulations and modification for targeted delivery enhance its appeal. However, intricate manufacturing processes, potential allergenic risks, and variability in biological responses among individuals pose challenges. pH and ionic strength influence encapsulation efficacy, and rapid clearance from the bloodstream may impact systemic immune responses. Despite these advantages, limitations in long-term stability and evolving clinical data require careful consideration. Studies have demonstrated the immunomodulatory effects of O.CS, such as increased production of proinflammatory cytokines and activation of immune cells^2,4,6,7^, suggesting that O.CS is a promising adjuvant for *A. paragallinarum* vaccines.

Silicon dioxide, or silica (SiO_2_), is a widely used nanomaterial due to its biocompatibility and low toxicity. SiO_2_ NPs provide an expansive surface area, improving stability and immunogenicity through enhanced antigen adsorption. The tunable pore structure allows controlled antigen release, influencing vaccine kinetics, while scalable production supports large-scale manufacturing. Challenges include intricate surface functionalization, potential immunogenicity, loading constraints, and environmental concerns. Compatibility issues with diverse antigens and formulations add complexity. However, limited in vivo data and long-term storage stability considerations further contribute to these challenges^8–11^. The physicochemical properties of SiO_2_, including its ability to adsorb antigens and protect them from degradation, make it an attractive candidate for the development of *A. paragallinarum* vaccines^2^. Additionally, surface-functionalized SiO_2_ nanoparticles can further modulate interactions with immune cells and enhance vaccine efficacy^8–11^.

Iron oxide (Fe_3_O_4_) offers inherent magnetic properties for targeted antigen delivery and controlled release, with easy incorporation into manufacturing processes. Potential imaging capabilities aid in tracking vaccine distribution. Fe_3_O_4_ NPs stimulate immune responses and allow for surface coating to improve stability. However, concerns about the biocompatibility, toxicity, and loading capacity limitations impacting vaccine efficiency have arisen. Potential interference with medical devices and challenges in biological fluid behavior pose additional considerations. Precision in manufacturing for targeted delivery requires specialized techniques, and rapid immune clearance may impact bloodstream persistence, with long-term biodistribution and clearance raising safety concerns^6,7^. Moreover, Fe_3_O_4_ nanoparticles can stimulate immune responses by activating immune cells and facilitating antigen presentation^12–14^. The distinctive physicochemical characteristics and immunomodulatory properties of Fe_3_O_4_ nanoparticles make them promising candidates for *A. paragallinarum* vaccines.

In summary, while chitosan-based adjuvants demonstrate adaptability and enhanced immune responses, they face challenges in terms of manufacturing complexity and potential allergenicity. Silicon-based adjuvants offer scalability but struggle with functionalization and compatibility issues. Iron oxide-based adjuvants present magnetic properties for targeted delivery but raise concerns regarding biocompatibility, toxicity, and manufacturing precision. Each has distinct advantages and drawbacks, emphasizing the importance of considering specific vaccine requirements and complexities in their application. The use of nanomaterials such as oleyl chitosan, silicon dioxide, and iron oxide in the vaccination of chicken against *A. paragallinarum* holds great potential for enhancing vaccine efficacy and the immune response. These nanomaterials offer unique properties, including improved antigen delivery, enhanced stability, and immunomodulatory effects. However, further research and development in this field are needed to optimize the formulation and utilization of these nanomaterials for effective infection control in poultry. This study aimed to prepare and study the antibacterial effects of several nanomaterials on *A. paragallinarum* and to use these nanomaterials as tools for vaccine improvement as adjuvants for infectious coryza vaccines by tracing the postvaccinal immune response against different serotypes of *A. paragallinarum.*

## Materials and methods

### Bacterial strains and culture

The *A. paragallinarum* reference strains, namely, the W strain (serovar A) and Modesto strain (serovar C), were obtained from MSD Animal Health/Intervet International in Boxmeer, The Netherlands. The reference strain 0222 (serovar B) was acquired from Dr. R.B. Rimler at the USDA National Animal Disease Center in Ames, Iowa, USA. The local field strain (serovar A) was originally isolated by the Anaerobic Vaccines Research Department at VSVRI from a laying flock in Egypt during an outbreak of Infectious coryza. The identification of the strain was confirmed through species-level identification and serotyping using serological tests with standard antisera against the reference serovars. To cultivate these strains, the plants were incubated under CO_2_ tension in a CO_2_ incubator at 37 °C for 24 h. The growth medium used was brain heart infusion (BHI) broth supplemented with 0.01% nicotinamide adenine dinucleotide (NAD). To ensure the purity of the bacteria, both aerobic and anaerobic cultures were examined on blood agar for any signs of contamination.

### Nanoparticles preparation

#### Synthesis of oleyl-chitosan (O.CS) nanoparticles

The Oleyl chitosan solution was prepared following the methods of Zlotnikov et al., with some modifications^15^. The procedure began by dissolving 5 g of chitosan in 2.5 L of 1% acetic acid solution. The resulting solution was stirred thoroughly overnight to ensure complete dissolution of the chitosan. Next, a solution of 5 g of oleic acid in 200 millilitres of ethanol was prepared. This oleic acid solution was added gradually in four portions to the chitosan solution while stirring continuously. The mixture was then stirred for an additional 30 min to ensure proper and uniform mixing of the components. Afterward, 1.37 g of EDC (ethyl-3-carbodiimide hydrochloride) was introduced into the solution, and stirring was continued at a temperature of 80 °C for 6 h. This step promoted chemical reactions between the various components. To adjust the pH, NaOH was used until a pH of 9 was achieved. After that, the solution was subjected to centrifugation at 5000 rpm for 30 min to separate the solid particles from the liquid. This centrifugation process was repeated three times, with each cycle involving washing the particles with water to remove any byproducts or impurities. Once the particles were thoroughly washed, they were dried using liquid nitrogen. This step aided in the removal of excess moisture, resulting in dried particles ready for further use. Finally, the dried particles were placed in a vacuum oven at 60 °C. The vacuum conditions in the oven facilitated the drying process, ensuring that the particles were completely dry and suitable for subsequent applications.

For the preparation of O.CS nanoparticles, 100 mg of O.CS was dissolved in a mixture of 60 ml of water, 2 ml of acetic acid, and 20 ml of ethanol. The flask was covered with parafilm to prevent evaporation, and the mixture was allowed to stir overnight. This extended stirring period ensured complete and uniform dispersion of the components. After overnight stirring, 20 mg of TPP (4 mg/ml DI-H_2_O) was added dropwise to the solution until it became slightly turbid. The solution was stirred for an additional 30 min to facilitate proper interaction and formation of nanoparticles. Once the stirring was complete, the solution was stored in a refrigerator. On the following day, the size and zeta potential of the particles were measured to evaluate the characteristics of the prepared O.CS nanoparticles.

#### Synthesis of silicon dioxide (SiO_2_) nanoparticles

SiO_2_ nanoparticles were prepared following the method described by Elzorkany et al.^16^, with some modifications. First, 45 ml of tetraethoxyorthosilicate (TEOS) was added to a 500 ml solution of 50% ethanol. The mixture was stirred for 30 min to ensure proper mixing. The pH of the solution was then adjusted to 11 by adding ammonium solution (25%). The adjustment was performed under vigorous homogenization at a high speed of 15,000 rpm. Before this step, the solution was continuously stirred overnight at 70 °C. Next, the washing process was carried out using DI-H_2_O. The solution was centrifuged at a speed of 10,000 rcf for 15 min, and this process was repeated until a neutral pH was achieved. This step was important for removing any impurities or unwanted residues. Following the washing process, the nanoparticles were dried in an oven at 80 °C overnight. This process ensured the complete removal of moisture, leaving behind dry particles. Finally, the dried SiO_2_ nanoparticles underwent calcination. The samples were heated in a furnace at a temperature of 550 °C for 4 h, at which point the temperature gradually increased at a rate of 5 °C per minute. Calcination helps to further stabilize and modify the properties of the nanoparticles.

#### Synthesis of SiO_2_-Chitosan (SiO_2_-CS) nanocomposites

SiO_2_-CS nanocomposites (NCs) were obtained with the method reported by Azhary et al., 2019, with modifications^17^. To prepare the SiO_2_-CS NCs, 1 g of chitosan and 4 g of SiO_2_ NPs were dissolved separately in 450 ml of 2% acetic acid solvent and stirred for 4 h until a clear solution was obtained (for the chitosan solution). SiO_2_ NP solution was added dropwise to the CS solution with stirring overnight. Thereafter, 330 mg of sodium tripolyphosphate (TPP) in 100 ml of DI-H_2_O solution was added to the previous solution and homogenized by a homogenizer at 10,000 rpm at room temperature for 30 min. To perform the physical characterization, the NCs were purified from any excess ligands through a triple centrifugation process. Centrifugation was conducted at 5000 relative centrifugal force (rcf) for 30 min at a temperature of 10 °C. After centrifugation, the resulting pellet containing the purified nanoparticles was carefully collected. It was then subjected to a drying process in an oven maintained at a temperature of 60 °C. The drying duration was 24 h, ensuring the complete removal of any remaining moisture. A portion of the SiO_2_-CS NCs solution containing the nanoparticles was kept separate for future use.

#### Synthesis of iron oxide (Fe_3_O_4_) nanoparticles

Fe_3_O_4_ NPs were prepared via hydrothermal synthesis using the coprecipitation method of Kandpal et al.^18,19^. Briefly, in a 250-ml three-necked flask under N_2_ gas, 5 g of iron (III) chloride hexahydrate and 1.84 g of iron (II) chloride tetrahydrate were dissolved in 71 ml of deionized water with stirring for 30 min. Next, 2.0 g of PVP was added to the solution as a stabilizer to control growth and particle agglomeration^20^. The pH of the solution was increased to 12 using 1 M NaOH with continuous stirring under N_2_ gas for 3 h. The prepared solution was repeatedly washed with an ethanol and distilled water mixture (1:1) via centrifugation at 5000 RCF for 30 min. Finally, the solution was dried in a vacuum oven at 100 °C for 24 h. The formation of Fe_3_O_4_ was confirmed via X-ray diffraction (XRD) spectroscopy.

### Nanoparticles characterization

To verify the uniformity, nanoscale size, and structure of the samples, various characterization techniques were employed. To examine the size and shape of the prepared nanoparticles (NPs), a small quantity of each sample was placed onto a copper grid and observed using a transmission electron microscope (TEM), specifically the Tecnai G20 model manufactured by FEI in the Netherlands. This approach allowed high-resolution imaging of the NPs. The morphology of the nanoparticles was described using a scanning electron microscope (SEM) (TESCAN VEGA COMPACT; TESCAN, Czech Republic). To confirm the phase and crystal structure of each sample, a small portion of the NPs was dried, and X-ray diffraction (XRD) patterns were obtained from the resulting dry powder. The XRD analysis was performed using a Philips Panalytical X’Pert-Pro instrument, and the patterns were obtained within the range of 2θ = 4°–80°. This analysis provided insights into the crystalline nature and phase composition of the samples. Furthermore, dynamic light scattering (DLS) and zeta potential measurements were conducted using a Zetasizer Nano ZS instrument manufactured by Malvern Instruments Ltd. in the UK. These measurements allowed for the determination of the hydrodynamic diameter, which provides information about the size distribution of the nanoparticles in a liquid medium. Additionally, the zeta potential, which relates to the surface charge of the particles, was also obtained. These measurements aided in understanding the stability and behavior of the samples in a colloidal suspension. By utilizing these characterization techniques, a comprehensive analysis of the samples' homogeneity, nanoscale properties, and structure was achieved, enabling a thorough understanding of their characteristics.

### Antimicrobial activity of the prepared nanoparticles

Formalin, which was added at a ratio of 0.25%, was utilized as an inactivating agent for the growth of *A. paragallinarum*^21^. The minimum inhibitory concentrations (MICs) of SiO_2_-CS (silicon dioxide with chitosan), O.CS (oleoyl-chitosan), SiO_2_ (silicon dioxide), and Fe_3_O_4_ (iron oxide) at different concentrations (200, 400, 600, and 800 µg/ml) were determined for the culture of *A. paragallinarum*. The culture mixture was incubated for 24 h at 37 °C, streaked into brain heart infusion (BHI) agar for confirmation and incubated at 37 °C for 24 h. The negative control shows a colony of *A. paragallinarum*.

### Confocal laser scanning microscopy

To assess the viability of *A. paragallinarum*, various concentrations (200, 400, 600, and 800 µg/ml) of the four different nanomaterials were tested. Confocal laser scanning microscopy (LSM 710, Carl Zeiss, Germany) was utilized as the imaging technique to determine bacterial viability. The evaluation of bacterial viability was performed by analyzing the live/dead ratio of the treated bacterial cells using a combination of propidium iodide (PI) and acridine orange (AO) stains. To conduct this analysis, 100 μl of *A. paragallinarum* bacteria was prepared at a concentration of 1 × 10^9^ CFU/ml in combination with 10 μl of the PI/AO mixture. The bacterial cells were then incubated in the dark with the PI/AO mixture for 15 min. After the incubation period, 50 μl of each sample was placed onto a coverslip to facilitate examination under a 40 × confocal laser scanning microscope. Acridine orange staining was used to selectively stain the live cells, which emitted green fluorescence upon excitation. On the other hand, propidium iodide was capable of penetrating only the membranes of dead cells, resulting in red fluorescence. By observing the fluorescence emitted by the stained bacterial cells, the live and dead cells could be distinguished, and their ratios could be quantified. This method provided valuable insights into the viability and survival of *A. paragallinarum* when subjected to different nanomaterial concentrations^22–25^.

### Vaccine preparation

To prepare cultures of *A. paragallinarum* serovars A, B, and C, the following steps were taken: The cultures were adjusted to a concentration of 1 × 10^9^ CFU/ml for each serovar^26^. Subsequently, equal volumes of each serotype were combined. The resulting serovar mixture was divided into five parts for the subsequent preparation of the inactivated vaccines. In the first part, the serovar mixture was adjuvanted with 400 µg/ml SiO_2_-CS, O.CS, SiO_2_, or Fe_3_O_4_ to create vaccines No. 1, 2, 3, and 4, respectively. The serovar combination was added dropwise to each nanocomposite (1v/1v) and vigorously shaken for one hour before being refrigerated until application. Finally, the fifth part was inactivated with 0.25% formalin^19^ and adjuvanted with mineral oil (liquid paraffin) following a modified method described previously^27^. Specifically, an emulsion was created with an aqueous phase-to-oil phase ratio of 30:70, resulting in vaccine no. 5, which is considered a traditional oil vaccine (positive control group). These preparations allowed the testing of different adjuvants and formulations with *A. paragallinarum* serovar A, B, and C cultures, enabling the evaluation of their potential for vaccine development.

### Quality control testing of the prepared experimental vaccines

#### Sterility test

The prepared vaccines were used directly after being tested for free from any contaminants, i.e., aerobic or anaerobic bacteria, fungi, or mycoplasmas, according to the World Organization for Animal Health^28^.

#### Safety test

The safety of the prepared vaccines was tested following the WOAH manual. Ten 21-day-old chicken were subcutaneously injected with a double field dose (2×) of the prepared vaccines to ensure that the vaccine was safe^29,30^. The inoculated chicken were observed for 14 days to detect any signs of local reaction due to *A. paragallinarum* symptoms^31^.

### Experimental design

This study involved several stages, including chicken maintenance, vaccination, blood sample collection, *A. paragallinarum* serology tests, challenge testing, and clinical symptom observation. To prevent interference from earlier immune responses, 180 specific pathogen-free chicken aged 4 weeks were obtained from the SPF farm Kom-Oshim in Fayum, Egypt. These chicken were housed in isolators and immunized against Newcastle, Mycoplasma, and Marek's diseases at the Veterinary Serum and Vaccine Research Institute (VSVRI) facilities. All the strains were confirmed to be free of *A. paragallinarum* infection and antibodies. The chicken were provided unrestricted access to feed without any antibacterial or anticoccidial components. The 180 chicken were divided into six groups, each comprising thirty chicken: Group 1: Infectious coryza vaccine adjuvanted with silicon dioxide with chitosan NCs (SiO_2_-CS). Group 2: Infectious coryza vaccine adjuvanted with oleoyl-chitosan NPs (O.CS). Group 3: Infectious coryza vaccine adjuvanted with silicon dioxide (SiO_2_). Group 4: Infectious coryza vaccine adjuvanted with iron oxide (Fe_3_O_4_). Group 5: Infectious coryza vaccine adjuvanted with mineral oil (liquid paraffin) (positive control). Group 6: Negative control group. These groups were established to evaluate the effectiveness of different vaccine formulations and adjuvants for infectious coryza, with a specific control group for comparison.

#### Vaccination

In the first, second, third, and fourth groups, the chicken were vaccinated with the mucosal nano vaccine (one drop was intranasal), and in the fifth group, the chicken were vaccinated with the traditional paraffin oil vaccine (0.5 ml S/C); the sixth group included control unvaccinated chicken and booster chicken 3 weeks after the first vaccination via the same route of administration.

#### Sampling

Blood samples were collected before vaccination, three weeks after vaccination (once per week), and three weeks after the booster (once per week). Subsequently, the serum samples were subjected to serological testing by tracing the antibody titer against different serotypes of *A. paragallinarum* after the first and booster vaccinations.

#### Evaluation of the humoral immune response in vaccinated chicken

The serum samples of all chicken were examined by the hemagglutinins inhibition (HI) test. Twofold dilutions of each serum sample were made. Hemagglutinins of the four Mexican isolates and strains were used, with antigen concentrations adjusted to 4 × HA units. The serum HI antibody titers are expressed as the reciprocal of the highest dilution of the serum sample that completely inhibited hemagglutinating activity^32–34^.

#### Evaluation of the immune response of vaccinated chicken through challenge methods

The challenge test was performed by intrasinus inoculation of 200 µl of broth culture containing the *A. paragallinarum* strains on day 42. The challenge dose was approximately 6 × 10^8^ cfu/ml, and each strain was individually tested^35^. Clinical symptoms were recorded daily and included sneezing, nasal exudate, facial swelling, snoring, and conjunctivitis, as previously described^36^. The results of the observations are based on both the presence and severity of clinical symptoms. A protected chicken was defined as a chicken that had shown no clinical signs.

### Statistical analysis

Antibody titers from the three immunized groups are expressed as the means. The variations within and between groups were analyzed using one-way and two-way ANOVA and Dunnett's multiple comparisons test in GraphPad Prism software version 8.0.2 for the samples^37^. The p values from the study were utilized to determine the significance of the observed differences. A p-value of less than 0.05 indicated statistical significance. A significance criterion of p < 0.05 was used, and significance levels are labeled in the graphs as follows: *p < 0.0332, **p < 0.0021, ***p < 0.0002, and ****p < 0.0001 for easier comprehension of the statistical data.

### Ethics approval and consent to participate

The experiments were approved by and adhered to the ethical standards outlined by the Institutional Animal Care and Use Committee at the Central Laboratory for Evaluation of Veterinary Biologics, with approval code (ARC 62429/11429). The experiments complied with bioethical standards and followed the American Research Institute (ARRIVE) guidelines.

## Results

### Characterization of nanoparticles

**Figure 1:**
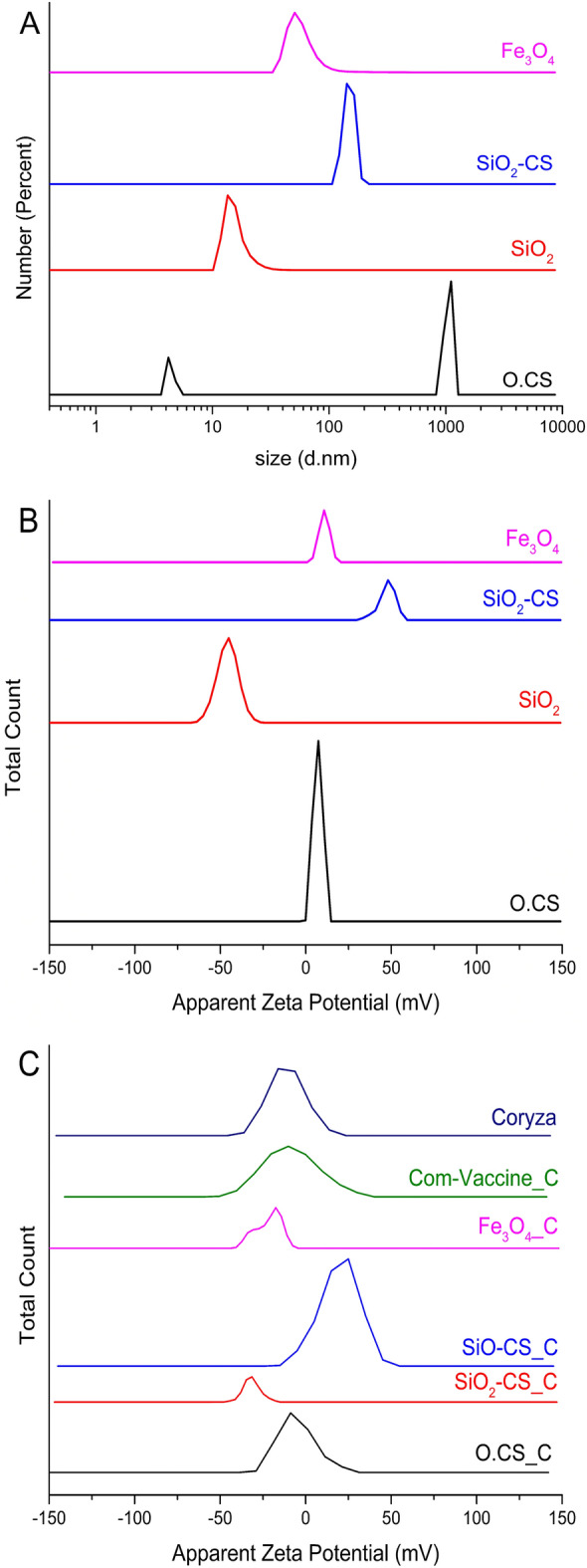
(**A**) the size distribution of the hydrodynamic diameter (in nm) of O.CS, SiO_2_, SiO_2_-CS, and Fe_3_O_4_ NPs determined via dynamic light scattering (DLS), as well as (**B**) their zeta potentials, and (**C**) zeta potentials of coryza (C) inactivated with commercial vaccine (Comm-Vaccine_C), O.CS_C, SiO_2__C, SiO_2_-CS_C, and Fe_3_O_4__C NPs.

The properties of nanomaterials can be tuned as desired by precisely controlling their stability, size, shape, synthesis conditions, and appropriate functionalization. In fact, the surface charge and stability of the prepared nanoparticles were characterized using zeta potential. The hydrodynamic diameter distributions of the O.CS, SiO_2_, SiO_2_-CS, and Fe_3_O_4_ NPs measured via dynamic light scattering (DLS) are presented in Fig. 1A. The O.CS, SiO_2_, SiO_2_-CS, and Fe_3_O_4_ NPs had hydrodynamic diameters of 1054 ± 71.89, 15.64 ± 3.6, 149.4 ± 15.77, and 59.01 ± 26.97 d. nm, respectively. The O.CS, SiO_2_, SiO_2_-CS, and Fe_3_O_4_ NPs had polydispersity index (PDI) of 0.87, 0.38, 0.74, and 0.50, respectively. The PDI is a measure of the distribution of particles sizes in a sample. This approach provides information about the uniformity or heterogeneity of the particles size distribution. A low PDI indicates a narrow size distribution, while a high PDI suggests a broader range of particle sizes.

The zeta potential of the prepared nanoparticles was measured and is presented in Fig. 1B. The O.CS, SiO_2_, SiO_2_-CS, and Fe_3_O_4_ NPs had zeta potentials of 7.14 ± 2.67, − 45.8 ± 6.14, 47.5 ± 4.82, and 9.13 ± 3.11 mV, respectively. The zeta potential of the prepared nanoparticles generally proves their stability in solution. It is important to highlight that nanoparticles are deemed stable when their zeta potential exceeds ± 30 mV, signifying the development of a strong electric bilayer encompassing the nanoparticles. This occurrence plays a crucial role in extending the stability of the material, broadening the scope of applications, especially in biological domains, by ensuring enduring stability over an extended period. The zeta potential of coryza (C) inactivated with nanoparticles and commercial vaccine (liquid paraffin) was measured and is presented in Fig. 1C. The coryza, comm-Vaccine_C, O.CS_C, SiO_2__C, SiO_2_-CS_C, and Fe_3_O_4__C had zeta potentials of − 11.0 ± 10.4, − 8.37 ± 15.9, − 5.05 ± 10.5, − 31.6 ± 5.14, 19.7 ± 11.3, and − 21.9 ± 7.41 mV, respectively.

**Figure 2:**
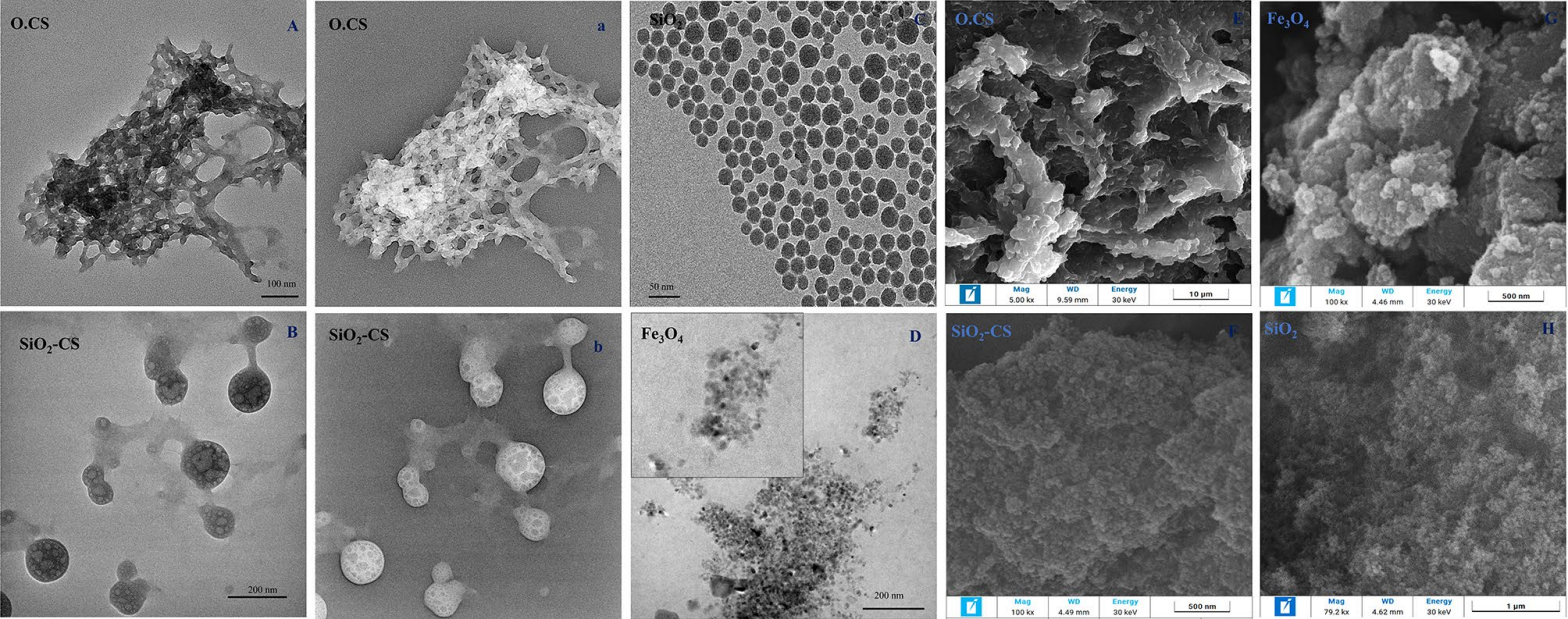
Transmission electron microscopy (TEM) images display the prepared nanoparticles, including O.CS nanoparticles shown in both an overall view (**A**) and –ve window view (a), SiO_2_-CS nanoparticles displayed in an overall view (**B**) and –ve window (depicting images with reverse grayscale) to observe a close-up view (b), SiO_2_ nanoparticles (**C**), and Fe_3_O_4_ nanoparticles (**D**) and a close-up view. (**E**–**H**) The morphology of the synthesized samples was confirmed by SEM analysis.

The nanostructure information and sizes of the prepared nanoparticles were observed via TEM. TEM was used to analyze the inner structure and features, such as the size, morphology at the atomic scale, and surface properties. TEM images (Fig. 2) show the morphologies of O.CS, SiO_2_, SiO_2_-CS, and Fe_3_O_4_ NPs prepared under different conditions. The TEM image of O.CS. shows physical aggregation of the chitosan nanoparticles with a roughly amorphous nature. SiO_2_-CS particles are almost spherical, with average diameters in the range of 100–170 nm. However, some of the particles were not spherical, and some of the SiO_2_ NPs were encapsulated in the CS. Additionally, SiO_2_ NPs have a nanoparticle appearance with average diameters in the range of 20–26 nm. Moreover, the Fe_3_O_4_ NPs exhibited two different morphologies: a spherical shape with a diameter of 30–90 nm and a square shape. The morphology of the synthesized samples was confirmed by SEM analysis, which was employed as a confirmatory examination for the synthesis of spherical-shaped nanomaterials **(**Fig. 2E–H). The SEM examination results obtained clearly demonstrate the spherical shape of the nanomaterials. However, because Olic acid is present in O.CS NPs, the O.CS also represents the polymeric network that maintains hardness.

**Figure 3:**
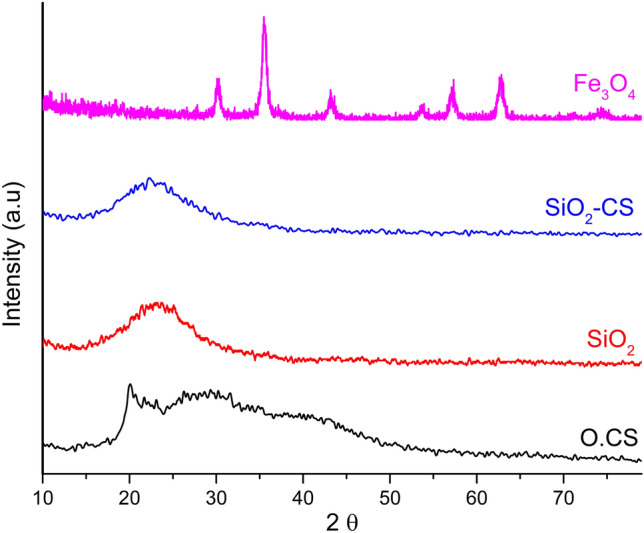
X-ray diffraction (XRD) patterns illustrating the characteristics of O.CS, SiO_2_, SiO_2_-CS, and Fe_3_O_4_.

The X-ray diffraction patterns of O.CS, SiO_2_, SiO_2_-CS, and Fe_3_O_4_ are shown in Fig. 3. The most vital reflection was noted at 21° for the O.CS NPs. A broad peak, which indicates the semicrystalline structure of O.CS NPs, is observed in the 24 to 50° range. This broad peak, with the highest intensity occurring at 29.37°, represents the semicrystalline nature of the O.CS NPs. XRD analysis of the SiO_2_ NPs revealed that the characteristic diffraction broad peak of SiO_2_ centered at 23° confirmed its amorphous nature. From the diffractogram, the most prominent amorphous peak is observed at 23° for SiO_2_-CS, which shows the amorphous state of the silica network with chitosan. Finally, diffraction peaks corresponding to the [220], [311], [422], [511], and [440] planes of the cubic Fe_3_O_4_ lattice appeared at 2θ values of 30°, 35.4°, 43°, 53.4°, 56.9°, and 62.5°, respectively. These peaks indicate a cubic inverse spinel structure of Fe_3_O_4_.

### Nanomaterials as inactivators

**Figure 4:**
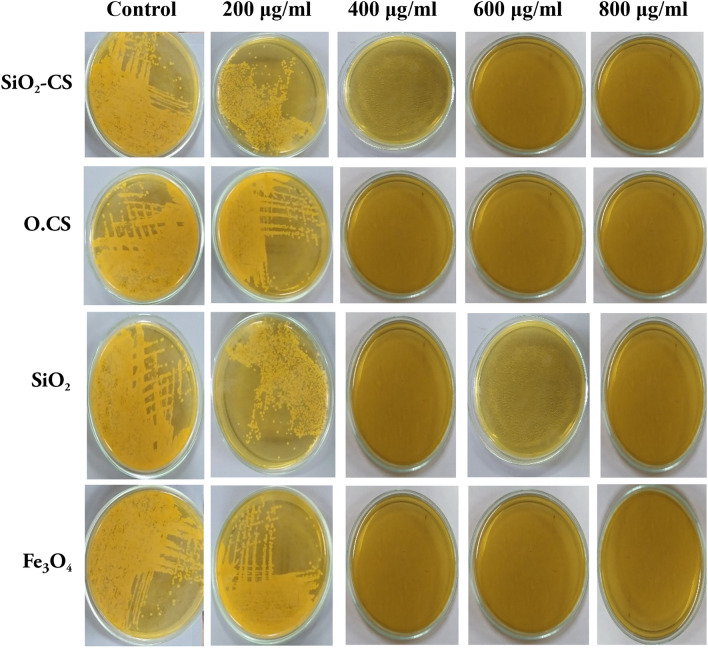
Results of the inactivation effect of different concentrations of O.CS, Si_2_O, SiO_2_-CS, and Fe_3_O_4_ nanomaterials on A. paragallinarum growth cultured in brain heart infusion agar (BHI).

As shown in Fig. 4, compared with the negative control, all the tested nanomaterials had synergistic effects on the greatest percentage of *A. paragallinarum* growth at a concentration of 400 µg/ml.

### Confocal live/dead imaging

**Figure 5:**
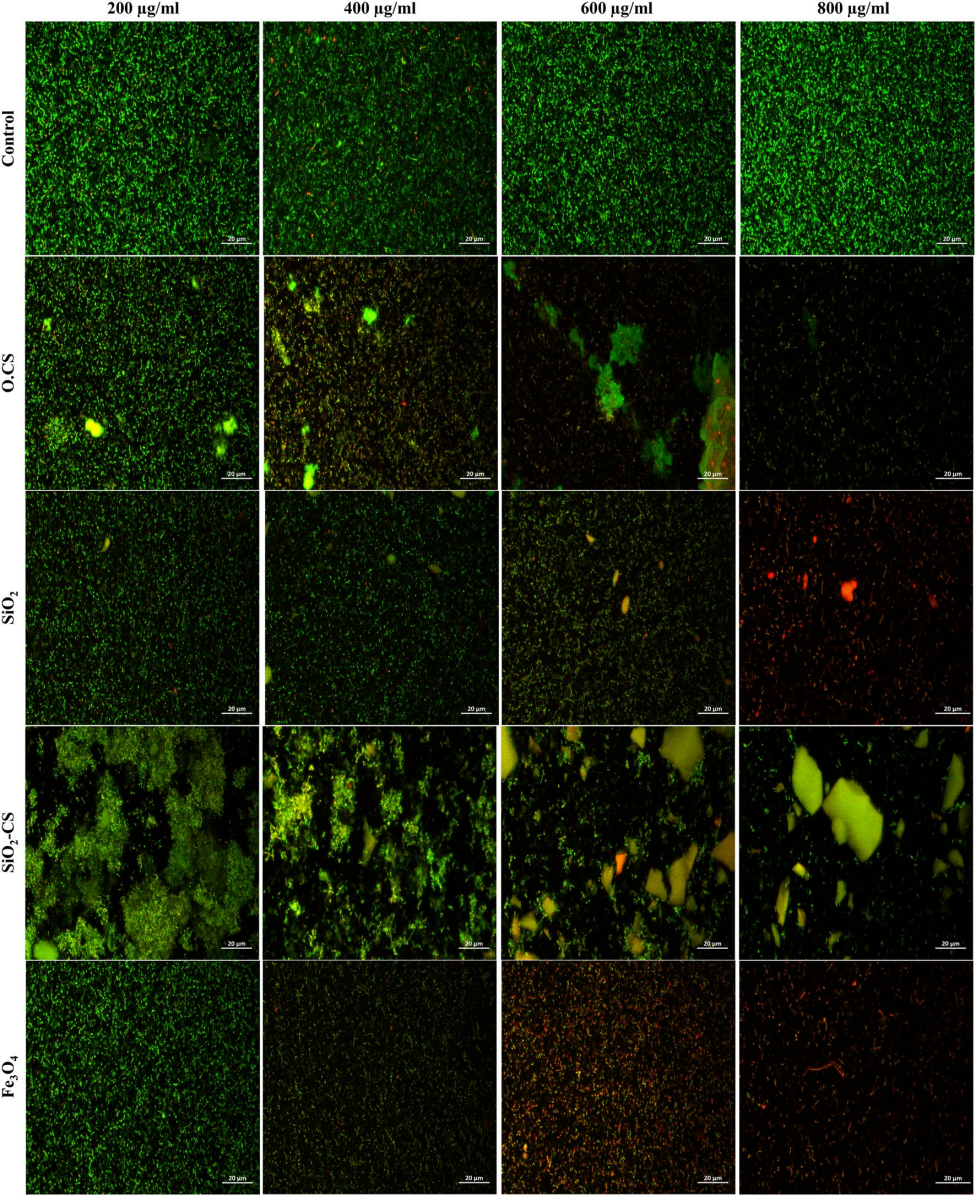
Confocal images of live and dead *A. paragallinarum* stained with AO and PI, respectively (scale bar = 20 µm).

**Figure 6:**
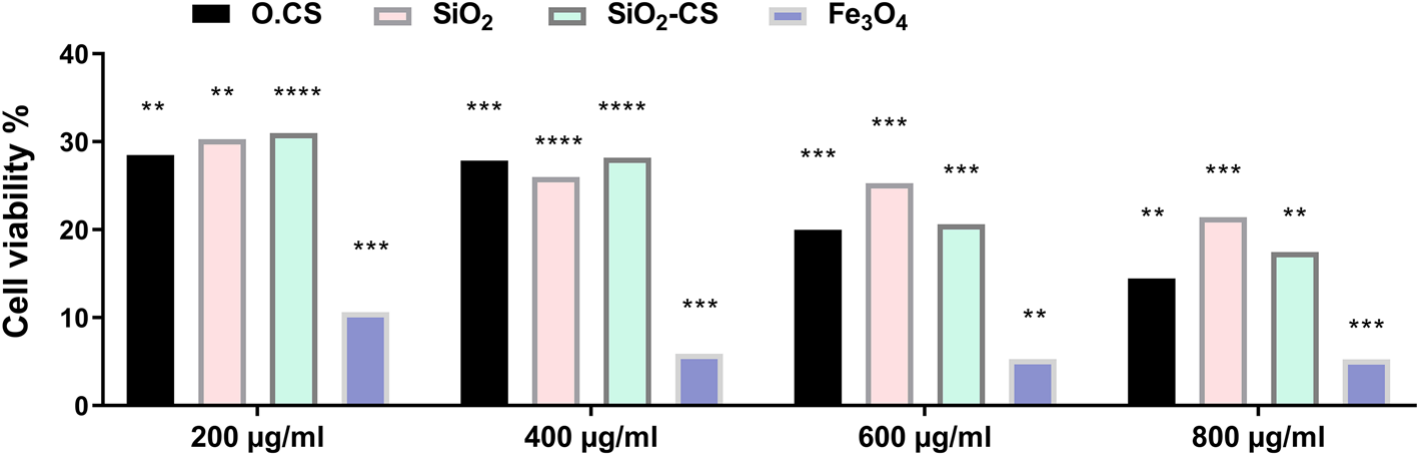
Cell viability percentage of *A. paragallinarum* after incubation with different concentrations of O.CS, SiO_2_, SiO_2_-CS, and Fe_3_O_4_ for 24 h. Statistically significant differences were *p < 0.0332, **p < 0.0021, ***p < 0.0002, and ****p < 0.0001.

The viability of live/dead *A. paragallinarum* was assessed after incubation with different concentrations of the four prepared nanomaterials separately using confocal microscopy imaging, as presented in Fig. 5. The images show that viable cells stained with AO emitted a green signal. In contrast, dead cells stained with PI emitted a red signal. O.CS, SiO_2_, SiO_2_-CS, and Fe_3_O_4_ NPs, especially Fe_3_O_4_ NPs, had significant cytotoxic effects on bacterial cells. The cell viability in culture media (control), O.CS, SiO_2_, SiO_2_-CS, and Fe_3_O_4_ NPs at different concentrations was evaluated and is shown in Fig. 6. As presented in Fig. 5, the cell viability percentage data indicate that significant cytotoxic effects were observed at different concentrations of all the prepared nanoparticles. The viability of the cells treated with Fe_3_O_4_ NPs reached nearly 5% at 3D and 4D relative to the control (untreated cells), indicating the remarkable cytotoxic effect of Fe_3_O_4_ NPs on *A. paragallinarum.*

### Quality control testing of the prepared experimental vaccines

Sterility test: No growth developed in media inoculated with different prepared vaccines. Safety test: At the end of the safety study, all the chicken were healthy and alive, no adverse reactions were observed, and there was no change in water or feed intake.

### Humeral immune response of vaccinated chicken to *A. paragallinarum* serovars

**Figure 7:**
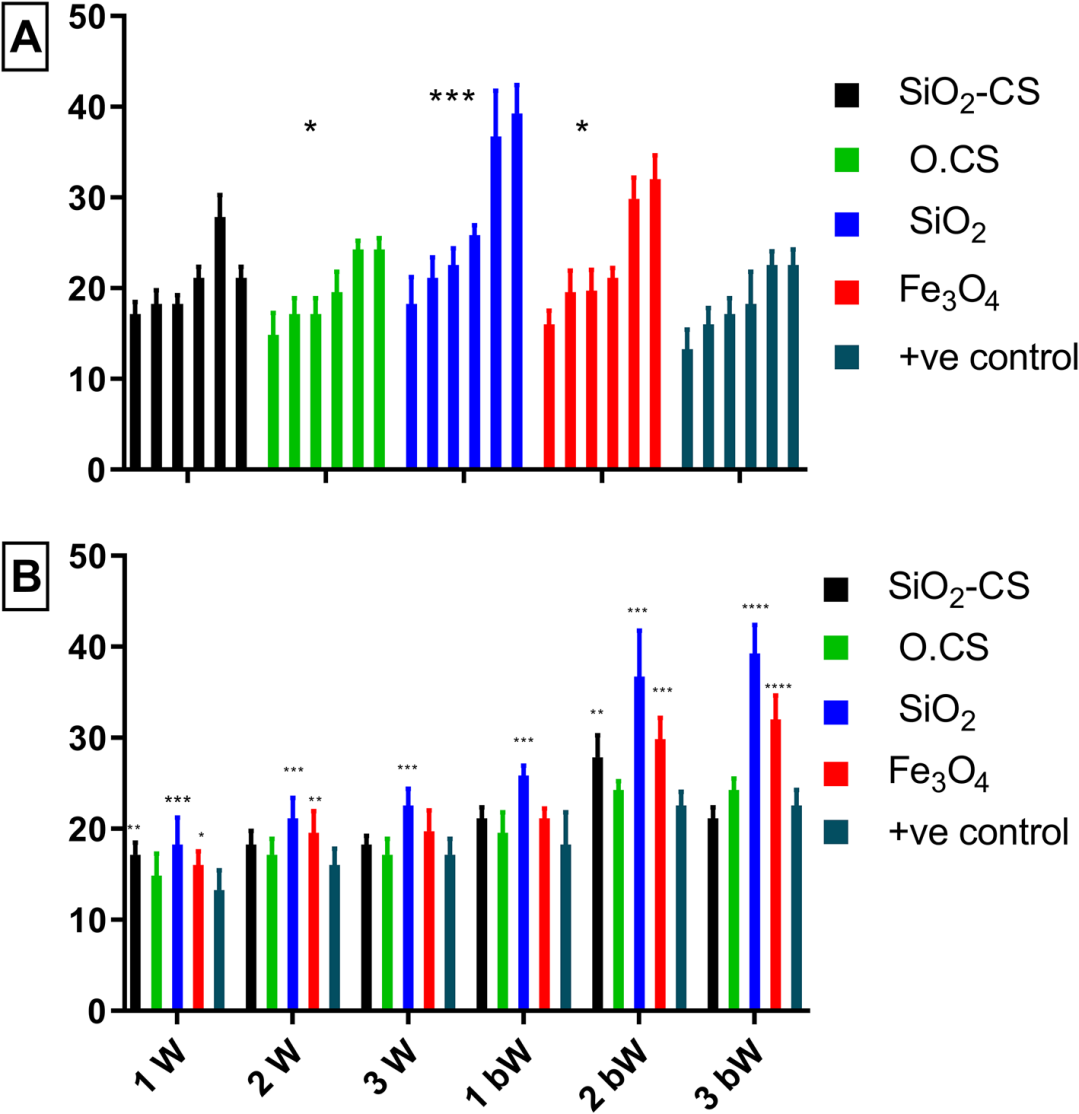
Geometric mean hemagglutinating antibody titers against A. paragallinarum serovar A in chicken sera after vaccination with O.CS, SiO_2_, SiO_2_-CS, and Fe_3_O_4_ nanovaccines and the + ve control vaccine. The (**A**) graph displays data pertaining to each vaccine throughout the experiment, whereas the (**B**) graph illustrates data for nanovaccines over each week. Statistical analysis, including both one- and two-way ANOVA, was performed to compare all nanovaccines against the + ve control group. Significance levels are denoted as *p < 0.0332, **p < 0.0021, ***p < 0.0002, and ****p < 0.0001. The term "bW" refers to the postboostering week.

Dunnett's multiple comparisons test is a statistical technique used to compare the means of several groups with a control group concerning *A. paragallinarum* serovar A. As shown in Fig. 7A, the test compares the control group to the following groups: SiO_2_-CS, O.CS, SiO_2_, Fe_3_O_4_, and + ve control. All of the comparison groups exhibited a markedly greater immunological response than the control group. Overall, these results suggest that the comparison groups had significantly different mean values than did the control group. The differences in mean values are statistically significant. The efficiency of the nanovaccines was ranked as follows: SiO_2_, Fe_3_O_4_, and O.CS demonstrated better efficiency than did the + ve control, with SiO_2_ being the most efficient. The SiO_2_-CS nanovaccine behaved similarly to the + ve control. In the 1st week postvaccination (Fig. 7b), the mean concentrations of SiO_2_, SiO_2_-CS, and Fe_3_O_4_ significantly differed from those in the + ve control group. However, the mean differences from O.S. are statistically insignificant. In the 2nd and 3rd weeks, the mean SiO_2_ concentration significantly differed from that of the + ve control group. However, the mean differences in SiO_2_-CS and O.CS are statistically insignificant. However, Fe_3_O_4_ had significant mean differences only in the 2nd week. After moving on to the "1st bW", "2nd bW", and "3rd bW" weeks, the mean difference in the SiO_2_ concentration was significantly different from that of the positive control. Fe_3_O_4_ showed significant mean differences at the 2nd bW and 3rd bW weeks. SiO_2_-CS, on the other hand, showed significant mean differences between the 1st bW and 2nd bW weeks. However, the difference from the mean O.CS was not statistically significant at these time points. It is worth noting that the level of significance varies among groups.

**Figure 8:**
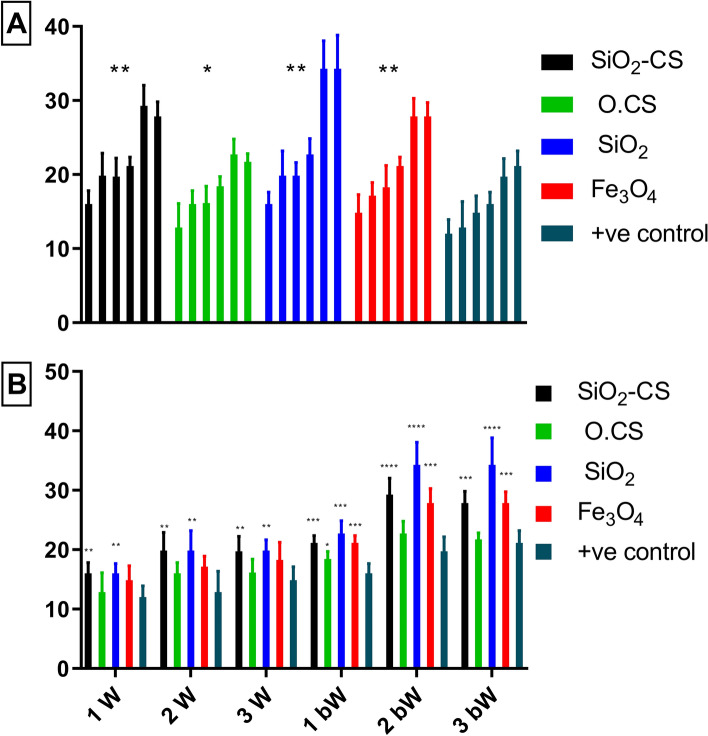
Geometric mean hemagglutinating antibody titer against *A. paragallinarum* serovar B in the sera of chicken vaccinated with O.CS, SiO_2_, SiO_2_-CS, and Fe_3_O_4_ nanovaccines and the + ve control vaccine. The (**A**) graph displays data pertaining to each vaccine throughout the experiment, whereas the (**B**) graph illustrates data for nanovaccines over each week. Statistical analysis, including both one- and two-way ANOVA, was performed to compare all nanovaccines against the + ve control group. Significance levels are denoted as *p < 0.0332, **p < 0.0021, ***p < 0.0002, and ****p < 0.0001. The term "bW" refers to the postboostering week.

**Figure 9:**
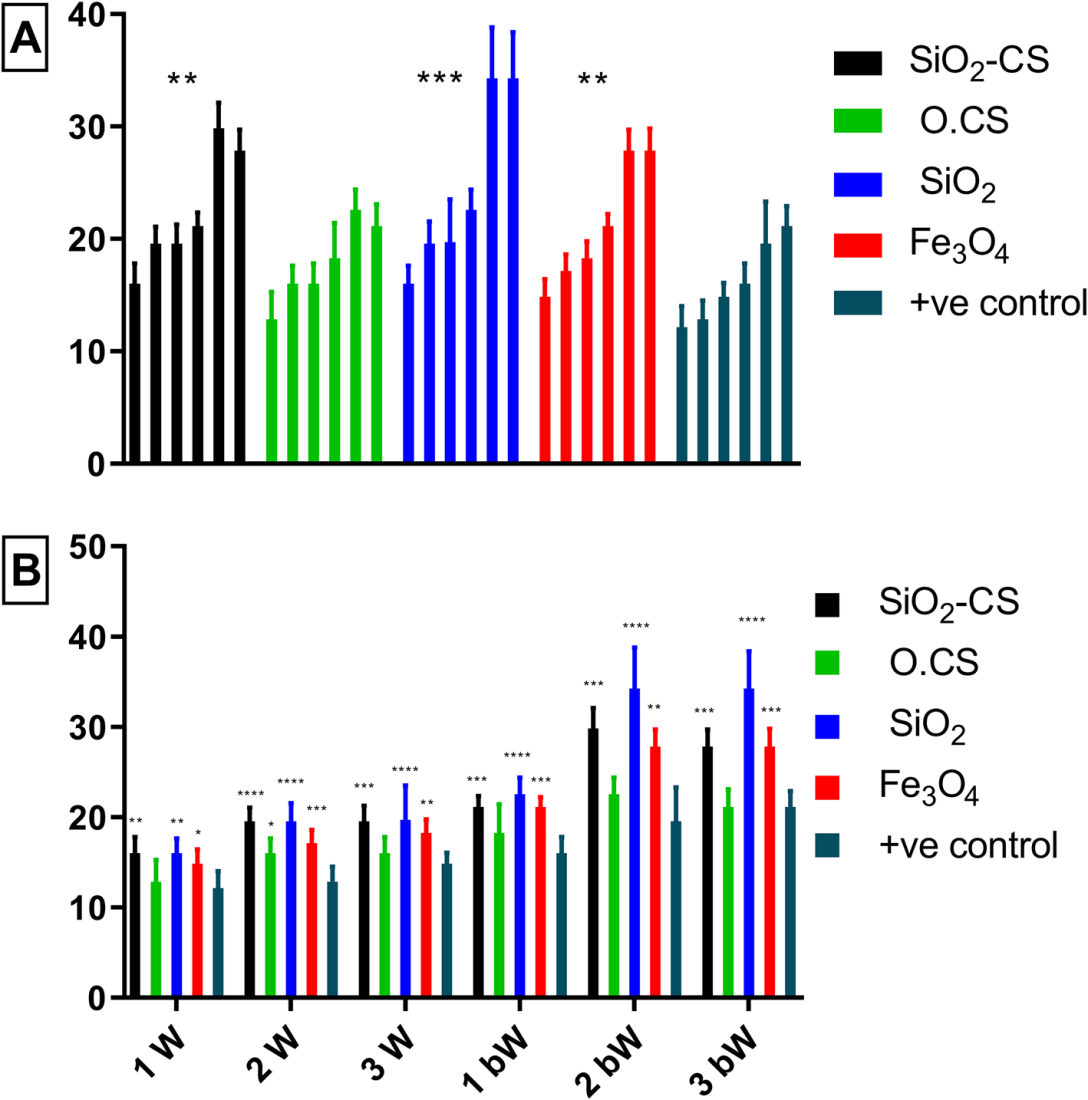
Geometric mean hemagglutinating antibody titer against A. paragallinarum serovar C in the sera of chicken vaccinated with O.CS, SiO_2_, SiO_2_-CS, and Fe_3_O_4_ nanovaccines and the + ve control vaccine. The (**A**) graph displays data pertaining to each vaccine throughout the experiment, whereas the (**B**) graph illustrates data for nanovaccines over each week. Statistical analysis, including both one- and two-way ANOVA, was performed to compare all nanovaccines against the + ve control group. Significance levels are denoted as *p < 0.0332, **p < 0.0021, ***p < 0.0002, and ****p < 0.0001. The term "bW" refers to the postboostering week.

Comparisons of means between groups and the control group in relation to *A. paragallinarum* serovars B and C. Figures 8A and 9A, show that all of the compared groups have a strong immunological response when compared to the control group. The observed variations in the mean values are statistically significant. The efficiency of the nanovaccines was ranked as follows: SiO_2_, Fe_3_O_4_, SiO_2_-CS, and O.CS. These combinations demonstrated better efficacy than did the + ve control, with SiO_2_ being the most efficient.

Figure 8B shows that the SiO_2_-CS and SiO_2_ groups had significant differences compared to the + ve control group in the 1st, 2nd, and 3rd weeks. However, there was no discernible difference between the + ve control and the O.CS groups. In all the 1st, 2nd, and 3rd weeks of comparison, the SiO_2_-CS, SiO_2_, and Fe_3_O_4_ groups differed significantly from the + ve control group. However, there was no substantial difference between the O.CS group and the control group at the 2nd or 3rd week. The difference was significant only for the 1^st^ bW band.

Figure 9B compares the means of the groups with those of the + ve control in relation to *A. paragallinarum* serovar C. The first set of comparisons in the 1st, 2nd, and 3rd weeks shows that the SiO_2_-CS and Fe_3_O_4_ groups exhibit substantial differences from the + ve control group. However, there was a significant difference between the + ve control and O.CS groups in the 2nd week. There was a significant difference between the + ve control and the SiO_2_ group comparisons in the 1st and 2nd weeks. In the 1st, 2nd, and 3rd bW comparisons, the SiO_2_-CS, SiO_2_, and Fe_3_O_4_ groups exhibited significant differences from the + ve control group. However, there was no significant difference compared with the O.CS group. Note that the absence of C-G6 in Figs. 7, 8, and 9 is intentional, as C-G6 serves as the negative control group with a consistent value of zero in the HI test. To avoid unnecessary clutter and enhance clarity, we did not include this control group in the figures.

### Challenge test

The effectiveness of four nanovaccine formulations containing different nanoadjuvants (vaccine no. 1, 2, 3, and 4) was compared to that of a commercial vaccine (vaccine no. 5), as summarized in Table 1. None of the chicken in any vaccinated group died during the entire challenge period. The chicken in the immunized groups were protected compared to those in the unimmunized control group (group 6). Within a week after the challenge, only a few chicken in the immunized groups displayed typical clinical signs of infectious coryza. In contrast, all chicken in the unimmunized control group (group 6) exhibited these signs.

**Table 1:** The results of the challenge test for the vaccinated and unvaccinated groups.

Chicken group	Vaccine No	Challenge strains	No. of chicken	No. of protected chicken	No. of unprotected chicken	Protection percentage %
SiO_2_-CS	1	W (serovar A)	10	8	2	80%
		0222 (serovar B)	10	8	2	80%
		Modesto (serovar C)	10	8	2	80%
O.CS	2	W (serovar A)	10	8	2	80%
		0222 (serovar B)	10	7	3	70%
		Modesto (serovar C)	10	7	3	70%
SiO_2_	3	W (serovar A)	10	9	1	90%
		0222 (serovar B)	10	8	2	80%
		Modesto (serovar C)	10	9	1	90%
Fe_3_O_4_	4	W (serovar A)	10	7	3	70%
		0222 (serovar B)	10	6	4	60%
		Modesto (serovar C)	10	7	3	70%
+ ve control	5	W (serovar A)	10	7	3	70%
		0222 (serovar B)	10	6	4	60%
		Modesto (serovar C)	10	6	4	60%
Control unvaccinated (−ve control)		W (serovar A)	10	0	10	0%
		0222 (serovar B)	10	0	10	0%
		Modesto (serovar C)	10	0	10	0%

The protection rates against strain serovar A were 80%, 80%, 90%, 70%, and 70% for vaccine no. 1, 2, 3, 4, and 5, respectively. For strain serovar B, the protection rates were 80%, 70%, 80%, 60%, and 60% for the corresponding vaccines. Similarly, for strain serovar C, the protection rates were 80%, 70%, 90%, 70%, and 60% for vaccine no. 1, 2, 3, 4, and 5, respectively. In contrast, the unvaccinated control group exhibited a 0% protection rate against all three serovars of *A. paragallinarum*.

## Discussion

Currently, in the veterinary field, safe and effective vaccines are powerful and effective tools for improving productivity and preventing infectious diseases. Most vaccines have been successfully used for many years; however, they have intrinsic limitations, such as variable efficacy and adverse effects. Moreover, nanomaterials are more effective adjuvants, and novel delivery systems may foster real vaccine effectiveness and timely implementation. Nanotechnology is accelerating the evolution of vaccines because nanomaterials have encapsulation ability and very advantageous properties due to their size and surface area and can serve as effective vehicles for antigen delivery and immunostimulatory agents. This approach is reflected in improving the performance of vaccines and thus protecting livestock from diseases. This study investigated the antimicrobial efficacy of several nanoparticles, such as O.CS, SiO_2_, SiO_2_-CS, and Fe_3_O_4_, against *A. paragallinarum* and their ability to improve the efficacy of the infectious coryza vaccine as an adjuvant in vaccine production.

The hydrodynamic diameter distribution, determined through dynamic light scattering (DLS), is another crucial parameter for understanding nanoparticle behavior in solution. In this study, the hydrodynamic diameters of the O.CS, SiO_2_, SiO_2_-CS, and Fe_3_O_4_ nanoparticles were measured (Fig. 2a). These measurements provide insights into the size distribution and dispersion of nanoparticles in the studied system^38–42^. The characterization of nanoparticles using zeta potential provides valuable information about their surface charge and stability. The zeta potential values obtained for O.CS, SiO_2_, SiO_2_-CS, and Fe_3_O_4_ nanoparticles in this study (− 45.8 to 47 mV) indicate their stability in solution (Fig. 1b). These findings align with previous findings in which zeta potential measurements were utilized to assess the stability of nanoparticles. Notably, nanoparticles with zeta potential values above 30 mV or below − 30 mV are considered stable, indicating the formation of a robust electric double layer surrounding the nanoparticles. This phenomenon contributes to prolonged stability, extending the application range, particularly in biological fields, as it ensures sustained stability over time^43,44^. The zeta potential of O.CS approaches zero, which is consistent with its underlying composition of chitosan and oleic acid. This process produces nanoemulsion particles with a nearly neutral charge and a small tendency toward a low positive charge. This particular combination contributes to the creation of a colloidal system with a slight positive charge, implying complex electrostatic interactions inside the nanoemulsion. The near-zero zeta potentials suggest that the O.CS and Fe_3_O_4_ nanoparticles have comparatively low surface charges, which could influence their behavior under different conditions and interactions with other biological entities. After coryza (C) underwent inactivation with nanoparticles and a local vaccine (liquid paraffin), the zeta potential data provides insightful observations regarding formulation stability and potential efficacy (Fig. 1c). The zeta potential of coryza (C) at − 11.0 ± 10.4 mV indicates a slightly negative surface charge, likely influenced by bacterial cell wall components and affecting interactions with nanoparticles. Similarly, the zeta potential of the positive group vaccine after inactivation (Comm-Vaccine_C) at − 8.37 ± 15.9 mV shows a negative charge with higher variability, possibly due to organic components. O.CS_C displays a similar slightly negative charge to coryza alone, suggesting minimal alteration by O.CS nanoparticles. Notably, SiO_2_-C and SiO_2_-CS_C exhibit significant changes, with SiO_2_-C's negative zeta potential implying enhanced stability through strong repulsive forces, while SiO_2_-CS_C's positive zeta potential may affect interactions with biological systems. Furthermore, Fe_3_O_4__C demonstrates a negative charge akin to the commercial vaccine. Overall, the post-inactivation zeta potential data elucidates how different nanoparticles impact the surface charge of inactivated coryza, thereby influencing stability, interactions with biological systems, and potentially formulation efficacy.

The TEM and SEM images presented in Fig. 2 reveal important information about the shape and size of the nanoparticles prepared under different conditions. For instance, the TEM and SEM image of O.CS nanoparticles indicates physical aggregation and a roughly amorphous nature. SiO_2_-CS nanoparticles exhibit mostly spherical shapes with average diameters ranging from 100 to 170 nm, although some nonspherical particles and encapsulated CS within the SiO_2_ NPs can be observed. SiO_2_ nanoparticles themselves appear as nanospheres with average diameters ranging from 20 to 26 nm. Additionally, the Fe_3_O_4_ NPs exhibited two distinct morphologies, one being spherical with a diameter of 30–90 nm and the other being square shaped. These observations are consistent with previous studies that utilized TEM and SEM to investigate the morphology of various nanoparticles^42,45–49^.

XRD analysis provides valuable information about the crystallographic structure of nanoparticles. Figure 3 confirms the structural characteristics of the O.CS, SiO_2_, SiO_2_-CS, and Fe_3_O_4_ nanoparticles. The sharp peak at 21° for the O.CS nanoparticles indicates a semicrystalline structure, while the broad peak at 27° suggests some degree of amorphous nature. SiO_2_ nanoparticles exhibit a diffraction peak centered at approximately 23°, confirming their amorphous nature. SiO_2_-CS nanoparticles also display a prominent amorphous curve at 23°, indicative of the amorphous state of the SiO_2_ network with chitosan. The diffraction peaks observed for the Fe_3_O_4_ nanoparticles suggest the cubic inverse spinel structure of the Fe_3_O_4_ nanoparticles^48,49^. These findings demonstrate the ability to tailor nanomaterial properties by controlling various parameters, as evidenced by the zeta potential, hydrodynamic diameter distribution, TEM images, and XRD patterns^2,6,7^.

The viability of *A. paragallinarum* was assessed using confocal microscopy after incubation with different concentrations of the prepared nanomaterials. Among the tested nanomaterials, the O.CS, SiO_2_, SiO_2_-CS, and Fe_3_O_4_ nanoparticles exhibited significant cytotoxic effects on bacterial cells, with the Fe_3_O_4_ nanoparticles showing the highest toxicity. The cell viability was evaluated for the control, O.CS, SiO_2_, SiO_2_-CS, and Fe_3_O_4_ nanoparticles at different concentrations, as shown in Fig. 5. The results indicated significant bactericidal effects at various concentrations of all the nanoparticles. Specifically, the viability of *A. paragallinarum* treated with Fe_3_O_4_ nanoparticles was reduced to nearly 5% at 600 and 800 µg/ml compared to that of the control, highlighting the remarkable bactericidal effect of Fe_3_O_4_ nanoparticles on *A. paragallinarum*.

The MIC was relatively high compared with that reported in previous works in which silica dioxide was studied, which was a limited effect. Research is still being conducted to determine the mechanism by which nanoparticles have an antibacterial impact. However, their mode of action differs from that of antibiotics. Because they have a larger surface area available for interactions than large particles, which boosts their bactericidal impact and causes cytotoxicity in bacteria, nanoparticles have greater antimicrobial activity. Akl 2020 suggested that these bacteria could work via one of the following mechanisms: compromising the bacterial cell envelope, inhibiting enzyme activity and DNA synthesis, producing reactive oxygen species through photocatalysis that damages bacterial cell components, or stopping energy transduction^50^. Additionally, it appears that the size of the microbe affects its cytotoxicity. Most bacteria have negatively charged cell membranes and have been shown to be the target of cationic biocides. One theory for the antibacterial activity of biocidal agents is their ability to penetrate the cell wall and engage in damaging interactions with the cytoplasmic membrane, which then allow intracellular components to seep out and ultimately cause cell death. In a recent study, mesoporous silica nanoparticles were shown to have antibacterial effects on both gram-positive and gram-negative microorganisms. The electrostatic interaction between the cationic head group of the mesoporous silica nanoparticles and the phosphate groups on the microbial cell wall was identified as the mechanism underlying the antibacterial activity of the particles.

Regarding the quality control testing of the prepared experimental vaccines in which each nanoparticle element (O.CS, SiO_2_, SiO_2_-CS, or Fe_3_O_4_) was used as an adjuvant in the formulation of the Infectious coryza vaccine in addition to the Paraffin oil adjuvant, the sterility test revealed no growth in different media inoculated with the vaccines. In the safety test, all the chicken were healthy and alive and exhibited no adverse reactions. There was no change in water or feed intake.

The humoral immune responses were measured in the five immunized groups using the HI test to determine the antibody titer. These findings could be attributed to the controllable particle size, large surface area, and easily modified surfaces owing to the presence of silanol groups (Si–OH), and the ability of NPs to interact with immunocompetent cells^8^. Interactions between NPs and the immune system have different outcomes that mostly depend on the characteristics of the NPs.

The vaccinated groups demonstrated greater protection than the unvaccinated control group. Each nanovaccine has a protection effect on different Av. paragallinarum serovars, representing the percentage of chicken that were protected from infection. This significant protection effect may be linked to the findings of Zhao 2016, who reported that SiO_2_ can mediate both cell-mediated and humeral immune responses in chicken^8^. These findings are consistent with those of Endang 2019, who concluded that Tetravalent coryza vaccination in chicken is effective at protecting against challenge with *A. paragallinarum* serovars A and B^35^.

The size and structure of NPs play important roles in determining how they interact with the immune system. Different shapes can influence how immune cells perceive and digest NPs. Smaller sizes (less than 50 nm) improve NP uptake without the need for passive or active transport. The aggregation of NPs can change how antigens are presented to immune cells, affecting the overall immune response. At noninhibiting or toxic concentrations, these aggregations may serve as scaffolds to enhance the growth of beneficial bacteria. O.CS, SiO_2_, SiO_2_-CS, and Fe_3_O_4_ NPs have considerable cytotoxic effects on bacterial cells, with Fe_3_O_4_ nanoparticles being the most toxic. These nanoparticles have the potential to function as antimicrobial agents against *A. paragallinarum*, presumably due to the role of iron in biological processes and protein synthesis.

SiO_2_ may act as an immunostimulant, boosting the immunological response of chicken to *A. paragallinarum*. Moreover, CS can influence the immunological response of chicken, potentially increasing both the innate and adaptive immune systems and facilitating effective resistance against *A. paragallinarum*. O.CS effectively wraps and distributes antigens, resulting in a stronger immunological response. Fe_3_O_4_ may activate immune cells, potentially leading to a particular and powerful immunological response to *A. paragallinarum*. The combination of chitosan and SiO_2_ NPs offers a multimodal strategy for treating coryza in chicken and controlling the immune response. SiO_2_ and Fe_3_O_4_ promote immunological activation, whereas CS and O.CS aid in immunomodulation and antigen delivery.

## Conclusion

The characterization of nanoparticles using zeta potential, hydrodynamic diameter distribution, TEM images, and XRD patterns provides valuable insights into their surface charge, stability, size, morphology, and crystallographic structure. The obtained zeta potential values indicate the stability of the O.CS, SiO_2_, SiO_2_-CS, and Fe_3_O_4_ nanoparticles. The hydrodynamic diameter distribution revealed the size distribution and dispersion of the nanoparticles. TEM images show the aggregation, amorphous nature, and different morphologies of the nanoparticles. XRD analysis confirmed the semicrystalline and amorphous structures of the O.CS and SiO_2_ nanoparticles, respectively, and suggested a cubic inverse spinel structure for the Fe_3_O_4_ nanoparticles. The prepared nanomaterials, particularly Fe_3_O_4_ nanoparticles, exhibited significant cytotoxic effects on *A. paragallinarum*, indicating their potential as bactericidal agents. The prepared experimental vaccines had no adverse effects and elicited immune responses against *A. paragallinarum*, with the SiO_2_ and SiO_2_-CS vaccines showing equal or greater efficacy than the commercial local vaccine (the positive control). Furthermore, the safety test confirmed the well-being of the vaccinated chicken. The use of nanomaterials as inactivators and adjuvants in the preparation of vaccines holds promise for controlling infectious coryza, a disease that causes substantial economic losses in the poultry industry. The use of SiO_2_, SiO_2_-CS, O.CS, and Fe_3_O_4_ NPs for treating coryza-infected chicken shows promise for modulating and enhancing immunological responses. These unique NPs may contribute to a more effective defense mechanism against *A. paragallinarum*, potentially leading to improved vaccine formulations and preventive strategies for coryza in poultry. However, further studies, including comprehensive immunological assessments and long-term efficacy evaluations, are warranted to establish the full potential of these nanoparticles in poultry health management.

## Data Availability

The data used during the current study are available from the corresponding author upon reasonable request.
